# Joint quantitative measurement of hTERT mRNA in both peripheral blood and circulating tumor cells of patients with nasopharyngeal carcinoma and its clinical significance

**DOI:** 10.1186/s12885-017-3471-6

**Published:** 2017-07-11

**Authors:** Xinsa Fu, Congxiang Shen, Huigang Wang, Fang Chen, Guanxue Li, Zhong Wen

**Affiliations:** Department of Otorhinolaryngology-Head and Neck Surgery, Zhujiang Hospital, Southern Medical University, Guangzhou, 510282 China

**Keywords:** Nasopharyngeal carcinoma, hTERT, Real-time quantitative PCR, Peripheral blood, CTCs

## Abstract

**Background:**

The study was aimed to quantitatively detect mRNA levels of the catalytic subunit of telomerase (hTERT) in both peripheral blood and circulating tumor cells (CTCs) of patients with nasopharyngeal carcinoma (NPC) and explore its significance in early diagnosis and treatment of NPC.

**Methods:**

hTERT mRNA levels in peripheral blood and CTCs of 33 NPC patients before and after treatment with intensity-modulated radiation therapy (IMRT) or/and chemotherapy and 24 healthy controls were measured using real-time quantitative PCR (qPCR) and their correlations to clinic pathological factors of NPC were analyzed.

**Results:**

Peripheral hTERT mRNA content was 10.75 ± 4.29 in NPC patients and 0.95 ± 0.37 in control subjects (*P* < 0.05), and had a significant correlation with patients’ clinical stage, T stage, and N stage (*P* < 0.05). Treatment of NPC patients at stages I and II with simple IMRT significantly reduced hTERT mRNA level from 5.60 ± 2.33 to 3.43 ± 1.42 (*P* < 0.05) and treatment of patients at advanced stage (III and IV) with induction chemotherapy followed by IMRT significantly reduced hTERT mRNA levels from 12.68 ± 3.08 to 10.68 ± 2.48 to 3.13 ± 1.69 (*P* < 0.05), respectively. In addition, the study also showed that hTERT mRNA content in CTCs of NPC patients was 10.65 ± 4.28, evidently higher than that of 1.09 ± 0.40 in control subjects (*P* < 0.05) and hTERT mRNA level in CTCs of NPC patients was obviously correlated to patients’ clinical stage, T stage and N stage (*P* < 0.05). After treatment, hTERT mRNA level in CTCs of NPC patients lowered from 10.65 ± 4.28 to 5.59 ± 2.32 (*P* < 0.05). The correlation analysis found that hTERT mRNA level in peripheral blood and CTCs of NPC patients were highly correlated with a correlation coefficient of 0.981.

**Conclusions:**

hTERT mRNA levels in peripheral blood and CTCs of NPC patients were significantly enhanced compared to that in healthy controls and highly correlated. Changes in hTERT mRNA level was closely correlated to patients’ clinical stage and T stage. Radiochemotherapy could effectively reduce hTERT mRNA level in peripheral blood and CTCs. Thus, it is possible using the joint detection of hTERT mRNA level in peripheral blood and CTCs as a new biomarker for early diagnosis, treatment efficacy and prognosis of NPC.

## Background

Nasopharyngeal carcinoma (NPC) is one of malignant tumors with high incidence in South China, especially in Guangdong Province [1, 2]. Currently, the etiology of NPC is still unclear. Although with advances in radiotherapeutical and chemoradiotherapeutical technologies, the 5-year survival rate of NPC patients at early stage has reached 70% [3], there is no effective and reliable means of early diagnosis. Clinically, most NPC patients are at advanced stage at admission, which undoubtedly greatly reduces the overall clinical cure rate of NPC. Thus, exploring early diagnosis technologies, particularly serology-based liquid biopsy and early diagnostic genetic technology, are extremely important for NPC patients. The only existing serological EB virus capsid antigen (VCA- IgA) and early antigen (EA-IgA) testing did not provide meaningful help for early diagnosis of NPC and could potentially increase patients’ economic and psychological burdens.

Liquid biopsy provides valuable indicators for early diagnosis, progression, efficacy evaluation and prognosis of tumor. Especially, peripheral blood diagnostic technology has become one of the most simple and effective diagnostic methods. Free RNA has been detected in the peripheral blood of patients with different tumors and the relationship of free RNA in peripheral blood of cancer patients has drawn more attention of many researchers. Some studies have showed that detection of free RNA in peripheral blood has higher sensitivity and tissue specificity than conventional tumor biomarkers [4–6], which opens a new field in tumor diagnosis, therapeutic evaluation and prognosis assessment. In recent years, many studies have detected hTERT mRNA in peripheral blood of patients with different tumors such as prostate cancer, lung cancer and stomach cancer [4, 5, 7] and demonstrated that elevated hTERT mRNA in peripheral blood is closely related with clinicopathological parameters, treatment efficacy and other tumor biomarkers of cancer patients.

With the deepening of cancer research, it was discovered that tumor cells could be released from primary tumor tissues into peripheral blood at the early stages, forming free CTCs. Thus, detecting CTCs is of great significance for detecting tumors at early stage, micrometastasis stage and recurrence stage, and for efficacy assessment, prognosis, and selecting right personalized treatment. Currently, detection of peripheral circulating tumor cells (CTCs) in recent years has become a research hotspot. Researchers have measured tumor-specific molecular markers using real-time quantitative PCR method in CTCs of patients with lung cancer, breast cancer, prostate cancer and other solid tumors and confirmed that the number of peripheral CTCs is related to early diagnosis of cancer patients as well as tumor metastasis and recurrence, and real-time monitoring peripheral CTCs is in favor of evaluating treatment efficacy and adjusting treatment modality [8–11]. Our previous researches have shown that 1) telomerase activity was enhanced in NPC patients at early stage, 2) this enhancement was closely associated with the clinical pathology of NPC, and 3) targeted inhibition of telomerase activity was capable of inhibiting NPC cell proliferation [12–14]. In this study, we further compared hTERT mRNA in peripheral blood as well as CTCs of NPC patients with that in peripheral blood of healthy controls using real-time quantitative reverse transcription polymerase chain reaction (qRT-PCR), analyzed its correlation with clinical and biological characteristics of NPC, and explored its clinical application value in NPC serological early diagnosis and treatment evaluation.

## Methods

### Clinical specimens and treatment

The study was approved by the Ethics Committee of our institute. All patients and their family members signed the informed consent form. Patients’ medical records were anonymous. 33 (16 male and 17 female) NPC patients diagnosed as undifferentiated squamous cell carcinoma at M0 at the Otolaryngology Development of Southern Medical University Zhujiang Hospital were enrolled in the study. They were at age of 23–70 years old with median age of 49 and staged according to the 2008 Clinical Staging System of NPC [15]. Among them, 4 were at stage I, 5 at stage II, 17 at stage III, 7 at stage IV, 11 at stage T1, 6 at stage T2, 13 at stage T3, 3 at stage T4,4 at stage N0, 5 at stage N1, 17 at stage N2, and 7 at stage N3. Patients at stages I and II were subjected to intensity modulated radiation therapy (IMRT) and patients at stages III and IV were subjected to two courses of induction chemotherapy followed by IMRT. Before treatment, after induction chemotherapy and after IMRT, 2 ml of peripheral blood was collected from each patient between 6:00 am to 9:00 pm. In addition, 24 non-cancer inpatients of the Otolaryngology Development of Southern Medical University Zhujiang Hospital were used as controls. For plasma isolation, peripheral blood of each patient and control subject was collected in an anticoagulated tube containing EDTA and centrifuged at 2000 rpm for 10 min within 2 h. The obtained plasma was stored at −80 °C. For CTCs isolation, peripheral blood was placed in a tube without EDTA, mixed with lymphocyte separation medium within 2 h, and centrifuged at 2500 rpm for 20 min. The obtained monocyte layer, which contains CTCs, was collected, washed with PBS and stored at −80 °C.

### Reagents and instruments

Trizol was from Invitrogen; PrimeScript RT reagent Kit and SYBR Premix Ex TaqII kit were from Takara; ABI7500 fluorescence quantitative PCR instrument was from ABI, USA; Ficoll-Hypaque lymphocyte separation medium was from Tianjin TCD.

### Primers

Primer sets for internal control β-actin (5′-TGACACCTCACCTCACCCAC-3′ and 5′-CACTGTCTTCCGCAAGTTCAC-3′) and primer sets for hTERT (5′-CGGAAGAGTGTCTGGAGCAA-3′ and 5′-GGATGAAGCGGAGTCTGGA-3′) were designed using Primer express and synthesized by Sangon Biotech (Shanghai) Co., Ltd.

### Real-time qRT-PCR

Two hundred fifty microliter of plasma was mixed thoroughly with 750 μL of Trizol and used for RNA extraction as described by the manufacturer. Total RNA concentration was measured using a UV spectrophotometer and RNA quality was determined by the ratio of absorbance at 260 nm and 280 nm. RNA samples with ratio of absorbance at 260 nm and 280 nm between 1.8 and 2.0 were considered to have high purity and stored at −80 °C for future use.

A total of 1 μg RNA of each sample was used for RT-PCR. cDNA was synthesized by reverse transcription as described by the manufacturer and amplified in a 20 μl of reaction system containing 10 μl of SYBR premix, 0.4 μl of each primer, 0.4 μl of ROX reference Dye II and 0.1 μl of cDNA template at the following reaction conditions: initial denaturation at 95 °C for 30s followed by 40 cycles of 95 °C for 15 s, 60 °C for 20s and 72 °C for 34 s. The melting curve of PCR was analyzed. The experiment was repeated three times and β-actin was used as an internal reference. Relative expression of hTERT to β-actin was calculated using 2^-△△Ct^.

### 1.5 statistical analysis

Statistical analysis was performed using SPSS13.0 software. Differences among multiple samples were compared using ANOVA. Differences in data obtained before and after treatments were analyzed using paired t-test and differences between two independent samples were analyzed using independent t-test. *P* < 0.05 was considered statistically significant.

## Results

### Peripheral hTERT mRNA level

#### Levels of peripheral hTERT mRNA in NPC patients and control subjects

The results showed that peripheral hTERT mRNA level was 10.75 ± 4.29 in NPC patients, significantly higher than that of 0.95 ± 0.37 in control subjects (*P* < 0.05), 9.17 ± 2.92 in NPC patients after induction chemotherapy (*P* < 0.05) and 2.66 ± 1.03 (*P* < 0.05) in NPC patients after IMRT (Table 1).

**Table 1:** Plasma hTERT mRNA level in NPC patients and control subjects

Group	n	hTERT mRNA (2^-△△Ct^) ^−^(X ± SD)	F value	*P* value
Control subjects	24	0.95 ± 0.37	−13.045	<0.001
NPC patients	33	10.75 ± 4.29		

#### Association of peripheral hTERT mRNA level in NPC patients with clinicopathological factors

Peripheral hTERT mRNA level was 0.95 ± 0.37 in control subjects, 3.35 ± 1.07 in NPC patients at stage I, 7.46 ± 0.91 in NPC patients at stage II, 11.17 ± 2.06 in NPC patients at stage III, 15.18 ± 2.93 in NPC patients at stage IV, 3.35 ± 1.07 in NPC patients at stage N0, 7.40 ± 0.95 in NPC patients at stage N1, 12.04 ± 3.15 in NPC patients at stage N2, 14.23 ± 2.44 in NPC patients at stage N3; 6.93 ± 3.34 in NPC patients at stage T1, 9.67 ± 2.66 in NPC patients at stage T2, 12.65 ± 1.72 in NPC patients at stage T3, and 18.67 ± 1.53 in NPC patients at stage T4, respectively, showing statistically significant differences (*P* < 0.05). In addition, the level of peripheral hTERT mRNA increased with the staging level increasing. Correlation analysis indicated that peripheral hTERT mRNA level in NPC patients was significantly related with clinical stage, tumor size and lymph node infiltration (*P* < 0.05), but not with gender (*P* > 0.05) (Table 2).

**Table 2:** Plasma hTERT mRNA level in NPC patients and its association with clinicopathological factors

Factor	No.	hTERT mRNA (2^-△△Ct^) ^−^(X ± SD)	F/t value	*P* value
Gender				
Male	16	10.48 ± 4.39	−0.346	0.731
Female	17	11.00 ± 4.31		
Age				
< 50 yrs	17	11.04 ± 4.84	0.395	0.696
≥ 50 yrs	16	10.44 ± 3.75		
Clinical stage				
I	4	3.35 ± 1.07	32.457	<0.001
II	5	7.46 ± 0.91		
III	17	11.17 ± 2.06		
IV	7	15.18 ± 2.93		
N Stage				
N0	4	3.35 ± 1.07	18.625	<0.001
N1	5	7.40 ± 0.95		
N2	17	12.04 ± 3.15		
N3	7	14.23 ± 2.44		
T Stage				
T1	11	6.93 ± 3.34	20.839	<0.001
T2	6	9.67 ± 2.66		
T3	13	12.65 ± 1.72		
T4	3	18.67 ± 1.53		

#### Relationship of peripheral hTERT mRNA level with the efficacy of IMRT or chemotherapy

A total of 9 NPC patients at stages I and II were directly subjected to IMRT and 24 NPC patients at stages III and IV were subjected to two courses of induction chemotherapy followed by IMRT. The result showed that peripheral hTERT mRNA level in all patients was significantly decreased after treatment (Tables 3 and 4).

**Table 3:** Plasma hTERT mRNA level in NPC patients at stages I and II before and after radiotherapy

Group	hTERT mRNA (2^-△△Ct^) (X ± SD)	*P* value
Before radiotherapy	5.60 ± 2.33	0.014
After radiotherapy	3.43 ± 1.42

**Table 4:** Plasma hTERT mRNA in NPC patients at stages III and IV before and after treatment

Group	n	hTERT mRNA (2^-△△Ct^) ^−^(X ± SD)	F value	*P* value
Before treatment	24	12.68 ± 3.08	98.528	<0.001
After chemotherapy	24	10.68 ± 2.48		
After chemotherapy and radiotherapy	24	3.13 ± 1.69		

### Level of hTERT mRNA in peripheral CTCs

#### hTERT mRNA level in peripheral CTCs of NPC patients and monocytes of control subjects

The results showed that hTERT mRNA level was 10.65 ± 4.28 in peripheral CTCs of NPC patients, significantly higher than that of 1.09 ± 0.40 in monocytes of control subjects (*P* < 0.05) (Table 5).

**Table 5:** hTERT mRNA level in peripheral CTCs of NPC patients and control subjects

Group	n	hTERT mRNA (2^-△△Ct^) ^−^(X ± SD)	t value	*P* value
Control subjects	24	1.09 ± 0.40	8.76	<0.001
NPC patients	33	10.65 ± 4.28		

#### Association of hTERT mRNA level in peripheral CTCs with clinicopathological factors of NPC patients

hTERT mRNA level in peripheral CTCs of NPC patients was significantly associated with tumors’ clinical stage, lymph node infiltration and metastasis, as well as distal metastasis (*P* < 0.05, Table 6). The results showed that hTERT mRNA level was 1.09 ± 0.40 in monocytes of control subjects, 3.65 ± 1.05 in peripheral CTCs of NPC patients at stage I, 7.18 ± 1.44 in peripheral CTCs of NPC patients at stage II, 10.97 ± 2.19 in peripheral CTCs of NPC patients at stage III and 15.13 ± 2.92 in peripheral CTCs of NPC patients at stage IV, showing significant differences among different subjects. In addition, hTERT mRNA level was 6.93 ± 3.14, 9.25 ± 3.03, 12.62 ± 1.78 and 18.85 ± 1.30 in peripheral CTCs of NPC patients at stage T1, T2, T3 and T4, showing no significant difference between the former two, but significant difference between the former two and the latter two (*P* < 0.05). Moreover, the later the stage was, the higher the hTERT mRNA level. Moreover, hTERT mRNA level was 3.65 ± 1.05, 7.18 ± 1.44, 11.86 ± 3.26 and 14.17 ± 2.43 in peripheral CTCs of NPC patients at stages N0, N1, N2 and N3, respectively, showing no significant difference between the former two, but significant difference between the former two and the latter two (*P* < 0.05).

**Table 6:** hTERT mRNA level in CTCs of NPC patients and its association with clinicopathological factors

Factor	No.	hTERT mRNA (2^-△△Ct^)^−^(X ± SD)	F/t value	*P* value
Clinical stage				
I	4	3.65 ± 1.05	28.95	<0.01
II	5	7.18 ± 1.44		
III	15	10.97 ± 2.19		
IV	9	15.13 ± 2.92		
N Stage				
N0	4	3.65 ± 1.05	16.36	<0.01
N1	5	7.18 ± 1.44		
N2	17	11.86 ± 3.26		
N3	7	14.17 ± 2.43		
T Stage				
T1	11	6.93 ± 3.14	20.83	<0.01
T2	6	9.25 ± 3.03		
T3	13	12.62 ± 1.78		
T4	3	18.85 ± 1.30		

#### Association of hTERT mRNA level in peripheral CTCs of NPC patients with the efficacy of chemotherapy and radiotherapy

A total of 9 NPC patients at stages I and II were directly subjected to IMRT and 24 NPC patients at stages III and IV were subjected to two courses of induction chemotherapy followed by IMRT. The results showed that hTERT mRNA level in peripheral CTCs of all NPC patients were significantly decreased after treatment (Table 7).

**Table 7:** hTERT mRNA level in CTCs of NPC patients before and after treatment

Group	n	hTERT mRNA (2^-△△Ct^)^−^(X ± SD)	*P* value
Before treatment	33	10.65 ± 4.28	<0.01
After treatment	33	5.59 ± 2.32	

### Association of peripheral hTERT mRNA level with hTERT mRNA level in peripheral CTCs of NPC patients

**Fig. 1:**
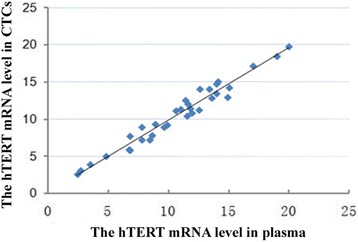
The scatter plot of hTERT mRNA level in plasma and CTCs of NPC patients

Peripheral hTERT mRNA content and hTERT mRNA level in peripheral CTCs of NPC patients were used to draw scatter plot and subjected to correlation analysis to obtain Pearson correlation coefficient. The results showed that all data were distributed near a straight line from the lower left to the upper right corner (Fig. 1) and had a Pearson coefficient of 0.981, showing a high correlation.

## Discussion

Early diagnosis of NPC has been the bottleneck in clinical treatment of NPC patients. Although there are many diagnostic techniques at the biological and clinical aspects, there is no early diagnosis of true meaning. Therefore, searching new technologies and methods for early diagnosis of NPC, especially from the aspects of peripheral serology and genetics is the key to the treatment of NPC.

### Detection of hTERT mRNA in peripheral blood of NPC and its possible clinical value

As we all know, telomerase as well as its catalytic subunit hTERT are closely related with the occurrence and development of vast majority of malignant tumors including NPC. Others and we [12, 16] have shown that telomerase was overexpressed in tumor tissues and tumor cell lines, but not in normal tissues and cells. However, the detection of telomerase activity in tumor tissues of patients who has been diagnosed is not helpful for early diagnosis of tumor. Directly detection of telomerase activity from peripheral blood may play an important role in early diagnosis of patients who has not been diagnosed by the naked eyes and microscopy. Miura et al. [17] detected hTERT mRNA in peripheral blood of liver cancer patients at early stage and compared it with other serological tumor markers AFP and DCP. Their results showed that serum hTERT mRNA content was higher in liver cancer patients than in patients with chronic liver diseases, but was not related with liver cancer differentiation level. The authors believed that hTERT mRNA in peripheral blood was superior in HCC diagnosis compared with AFP mRNA, AFP and DCP. In addition, they found that serum hTERT mRNA level was significantly different in different tissues and could be used as a new and useful biomarker for cancer. March et al. [18] also detected plasma hTERT mRNA content in 49 patients with prostate cancer and found plasma hTERT mRNA content was significantly higher in patients with locally advanced lesions than in patients with localized lesions. Thus, it can be used as a non-invasive marker of prostate cancer to differentiate patients with locally advanced disease at molecular level. Kang [7] and March-Villalba et al. [19] detected peripheral hTERT mRNA content in patients with gastric and prostate cancer, respectively. Their results showed that hTERT mRNA content in these cancer patients was higher than that in control subjects. They further found that hTERT mRNA content in peripheral blood of patients with gastric and prostate cancer was also closely associated with clinicopathological parameters such as clinical stage, lymph node infiltration range, etc., but not with age and gender. Our study showed for the first time that hTERT mRNA level in peripheral blood of NPC patients was higher than that in control subjects, and was also significantly correlated with patients’ clinical stage, tumor size and lymph node infiltration scope (*P* < 0.05). In addition, our results further showed that hTERT mRNA level in peripheral blood of NPC patients at early stage was markedly increased, and this increase became more obvious with severity of tumor stage: the later the stage (for both N and T staging systems) was, the higher the peripheral blood hTERT mRNA content, suggesting that this serological marker could have significance in early diagnosis and clinical staging severity assessment of NPC patients. Our results are consistent with the findings of other scholars on different cancer patients [6, 20–23].

Combined detection of hTERT mRNA with other diagnostic techniques showed a potentially significant value in improving the accuracy of tumor diagnosis and evaluating treatment efficacy of malignant tumors. Ping et al. [24] found that plasma hTERT mRNA detection combined with PDG-PET/CT can improve the accuracy of tumor diagnosis. Salvatore et al. [25] reported that for colorectal cancer patients receiving neoadjuvant chemotherapy, peripheral hTERT mRNA can be used to assess the efficacy of chemotherapy. Lu et al. [26] also showed that peripheral hTERT mRNA level in patients with acute myelogenous leukemia after first chemotherapy decreased to the level of 2.4 ± 2.0 at complete remission from that of 13.5 ± 8.5 before the first treatment (*P* < 0.001). Among them, most had normal hTERT mRNA level of 1.2 ± 0.8. Changes in peripheral hTERT mRNA content can also be used to assess the results of tumor surgery. Lu et al. [26] reported that the content of hTERT mRNA in peripheral blood of patients with laryngeal squamous cell carcinoma after surgery significantly reduced to 8.0 ± 5.7 from that of 11.8 ± 8.3 before surgery (*P* = 0.03), suggesting that peripheral hTERT mRNA may become an important indicator for observation of therapeutic responses of cancer patients.

Our results also showed that peripheral hTERT mRNA content of NPC patients at late stage (III and IV) reached 12.68 ± 3.08 before combined therapy, and was reduced to 10.68 ± 2.48 and 3.13 ± 1.69, respectively, after inductive chemotherapy as well as concurrent IMRT and chemotherapy. It can be seen from the results that after inductive chemotherapy, peripheral hTERT mRNA level was decreased slightly in most patients, while after concurrent IMRT and chemotherapy, hTERT mRNA level was significantly decreased in almost all patients. Moreover, after IMRT, hTERT mRNA level was decreased from 5.60 ± 2.33 to 3.43 ± 1.42 in NPC patients at early stages (I and II). All these results suggested that IMRT or concurrent IMRT and chemotherapy had more obvious impacts on peripheral hTERT mRNA level than induction chemotherapy, suggesting that NPC may be more sensitive to radiation therapy. In addition, the action duration of radiation therapy was also longer than that of chemotherapy, leading to more obvious inhibition of telomerase activity and tumor micrometastasis via blood in NPC patients, thereby affecting peripheral hTERT mRNA content. These results in turn support clinical treatment modalities of radiotherapy supplemented by chemotherapy for NPC patients [27].

### Detection of hTERT mRNA in peripheral CTCs of NPC patients and its clinical significance

The fact that examination of peripheral CTCs of cancer patients becomes a hot spot of research shows the importance of this examination. CTCs detection is considered as an important indicator of tumor diagnosis, distal micrometastasis, treatment efficacy monitoring and prognosis. However, due to the heterogeneity and rarity of CTCs, the use of a single marker is generally challenged with lower specificity and sensitivity. Because of the difficulty of selecting individual CTC, studies including ours [9] usually use peripheral monocyte layer as material for CTCs. Currently, most researchers use multiple genetic markers to identify peripheral CTCs with one marker positive considered as CTC positive [28]. The detection of hTERT mRNA in peripheral CTCs is equally important as detection of hTERT mRNA in peripheral blood for understanding the source of hTERT mRNA and early diagnosis of cancer. Wang et al. [29] used multiple markers including hTERT mRNA for detecting CTCs and found that 69.2% of blood samples of breast cancer patients were positive to at least one cancer-associated genetic marker and after completion of treatment, CTCs positive rate dropped to 20.6%.

Our study showed for the first time that hTERT mRNA level in peripheral CTCs was 10.65 ± 4.28, significantly higher than that in control subjects, and significantly correlated with clinical stage, tumor infiltration scope and lymph node metastasis. These results were similar to that in peripheral blood. Even in early stage NPC patients, hTERT mRNA in peripheral CTCs was also increased, and this increase became more obvious with the progress of clinical stage.

Increased peripheral CTCs and hTERT mRNA level are likely new evidences for distal micrometastasis. Cheng et al. [8] screened CTCs in peripheral blood and found CTCs count in peripheral blood was correlated with bone metastasis of lung cancer. For NPC patients, distal metastasis to bone, lung, liver or multiple organs is one of the main causes for treatment failure [30]. Timely detection of CTCs in peripheral blood and hTERT mRNA content may provide clinical assistance for determination of factors related to tumor recurrence and micrometastasis as well as early intervention of patients with metastasis. In this study, all included 33 patients with newly diagnosed NPC had no distal metastasis. Therefore, it is necessary to conduct controlled prospective study with a large sample size to clarify its relationship with distal metastasis and micrometastasis of NPC, and provide a new reference index for early diagnosis and treatment of NPC.

### Correlation analysis of hTERT mRNA in peripheral blood with CTCs

In this study, we for the first time simultaneously examined hTERT mRNA content in both peripheral blood and peripheral CTCs of NPC patients. The results showed that they increased consistently and appeared to have significant correlation, thus could be used as new serum biological indicators for early diagnosis of NPC. In addition, the results also showed that they were closely related to tumor clinical stage, size and lymph node infiltration scope as well as other clinicopathological factors, and reduced after radiotherapy and chemotherapy, suggesting that joint detection of these indicators could serve as one of the indicators for NPC progression, early peripheral micrometastasis, treatment efficacy and prognosis. These results also laid a foundation for further exploration of the source of peripheral hTERT mRNA and its underlying mechanism.

## Conclusions

In conclusion, The hTERT mRNA levels are frequently upregulated in peripheral blood and CTCs in the patients with nasopharyngeal carcinoma (NPC) and also the overexpression of hTERT mRNA correlated with clinicopathological parameters of NPC. Radiochemotherapy could effectively reduce hTERT mRNA levels in peripheral blood and CTCs in NPC. Thus, the joint detection of hTERT mRNA level in peripheral blood and CTCs may serve as a new biomarker for early diagnosis, treatment efficacy and prognosis of NPC.

## Abbreviations

CTCs: Circulating tumor cells
EA-IgA: Early antigen
hTERT: Human telomerase reverse transcriptase
IMRT: Intensity modulated radiation therapy
NPC: Nasopharyngeal carcinoma
qRT-PCR: Quantitative reverse transcription polymerase chain reaction
VCA- IgA: Virus capsid antigen
